# Editorial: Neurobiological Biomarkers for Developing Novel Treatments of Substance and Non-substance Addiction

**DOI:** 10.3389/fpsyt.2021.811032

**Published:** 2021-12-20

**Authors:** Yi Liu, Ling Li, Yunfei Wang, Dara G. Ghahremani, Jianhua Chen, Kyoji Okita, Wenbin Guo, Yanhui Liao

**Affiliations:** ^1^Department of Psychiatry, Sir Run Run Shaw Hospital, Zhejiang University School of Medicine, Hangzhou, China; ^2^National Clinical Research Center for Mental Disorders, Department of Psychiatry, The Second Xiangya Hospital of Central South University, Changsha, China; ^3^Department of Psychiatry & Biobehavioral Sciences, Semel Institute for Neuroscience and Human Behavior, University of California, Los Angeles, Los Angeles, CA, United States; ^4^Shanghai Mental Health Center, Shanghai Jiao Tong University School of Medicine, Shanghai, China; ^5^Integrative Brain Imaging Center of National Center of Neurology and Psychiatry, Kodaira, Japan

**Keywords:** neurobiological biomarkers, novel treatments, addiction, substance addiction, behavioral addiction

## Introduction

Many people suffer from substance (e.g., nicotine, alcohol, and heroin) or non-substance/behavioral (e.g., gaming, gambling, and social media) addiction (1–8). Both types of addiction are associated with numerous medical, economic and social consequences, resulting in a significant burden on individuals, families and society (3, 9–11). Unfortunately, the effectiveness of current treatments for either substance or non-substance addiction is largely insufficient. Detecting biomarkers and exploring new treatment strategies will help accelerate advancements in the field by providing novel targets for treating substance and non-substance addiction, detecting treatment subgroups, better-predicting treatment response, and improving overall outcomes. Thus, this Research Topic “*Neurobiological Biomarkers for Developing Novel Treatments of Substance and Non-substance Addiction*” arose. A total of 44 articles, including two commentaries, were included in this Research Topic. Among the 42 research articles, 33 articles were associated with substance addiction, eight articles focused on non-substance addiction, and one article involved the interaction of smoking and Internet Gaming Disorder (IGD) (see Table 1). All of articles focused on examining underlying neurobiological mechanisms of substance or non-substance addiction with the orientation toward determining effective treatments.

**Table 1:** Characteristics of the 42 publications.

**Category of addictions**	**Design**		**No articles**
Substance addiction	Drugs		
	Heroin	Clinical study, pre-clinical study	3
	Methamphetamine	Clinical study	7
	Opioid	Clinical study, pre-clinical study	3
	Benzodiazepine	Clinical study, review	2
	Ketamine	Pre-clinical study	1
	Sleep problems in drug users	Cross-sectional study	1
	Alcohol	Clinical study	8
	Tobacco	Clinical study	4
	Others		
	Betel-quid	Clinical study	2
	Substance use disorder	Review, meta-analysis	2
Non-substance addiction	Internet	Cross-sectional survey	3
	Internet game	Clinical study, cross-sectional survey	5
	Smart phone	Cross-sectional survey	1

## Substance Addiction

Chen, Qin et al. conducted a meta-analysis and provided evidence that transcranial direct current stimulation (tDCS) can be an effective way to reduce the craving of a substance or food, with greater reductions of craving resulting from longer multiple stimulus durations. Fuchshuber and Unterrainer reviewed psychoanalytic theories of substance use disorder (SUD) etiology and emphasized the importance of increased sadness and anger and proposed an improved substance use disorder (SUD) causal model, which assumed that childhood trauma influences the development of symptoms of SUD by the increase of personality structural defects and the tendency for sadness and anger.

### Illicit Drug Addiction

In the explorations of gender differences in sleep problems among drug users, He H. et al. conducted a cross-sectional study and found that in a group of individuals who use methamphetamine (MA), females reported a higher frequency of sleep problems and poorer sleep quality than males; this effect was not observed in those that use heroin or other drugs. The results suggest that future studies that aim to determine the effects of treatment interventions should not neglect the influence of sleeping problems and gender differences.

Luo et al. analyzed brain functional connectivity in 51 heroin-dependent (HD) individuals and 40 healthy controls (HCs) who underwent resting-state functional magnetic resonance imaging (rs-fMRI), examining the “amplitude of low-frequency fluctuation” (ALFF) using machine learning analyses (support vector machines, SVMs). The results suggested that the accumulative effect of heroin's neurotoxicity overpowered self-recovery of the brain and that the measure may be applied as a potential biomarker to distinguish HD individuals from controls. Huang J.-X. et al. examined the association between Buddhist belief and suicide risk in Chinese persons receiving methadone maintenance therapy (MMT) for heroin dependence. Mei et al. demonstrated that heroin and methamphetamine can be substituted for each other in terms of reinforcement effects in rats, providing rationale for exploration of a dose-effect mechanism of heroin and methamphetamine.

Liu, Luo, et al. explored genome-wide DNA methylation in two groups of individuals who use MA with different qualities to addiction (i.e., dependent or not based on DSM-IV criteria). They identified seven specific pathways with abnormal methylation status, suggesting that the circadian entrainment pathway and the caveolin-2 gene may play key roles in MA addiction quality. Yang, Zhao et al. assessed serum levels of interleukins (ILs) in methamphetamine-associated psychosis (MAP) and their relationships with psychotic symptoms and cognitive dysfunction. The results showed that serum levels of IL-6 and IL-8 were significantly greater in MAP patients, suggesting that immunological disturbances are related to MAP and that IL-2R, IL-6, IL-8, and IL-10 are associated with the severity of psychotic symptoms and cognitive function impairment. Wang, Zuo et al. revealed that impulsivity and various drug use characteristics can significantly predict quality of life (QOL) in MA use disorder (MUD) patients, and provided clinical guidance for the treatment of MUD patients. Yan et al. used machine learning methods to determine the most relevant graph theory-based features of functional connectivity of the brain that predict treatment success in MA-dependent participants; they provided potential biomarkers to differentiate and predict the treatment response for MA-dependent patients. Pilhatsch et al. conducted a 3-month longitudinal study on MUD patients. A probabilistic reversal learning (PRL) paradigm was implemented to uncover the microstructure of impulsive choice and maladaptive learning strategies in 23 patients with MUD in comparison with 24 controls. The study demonstrated behavioral correlates of maladaptive decision-making processes in patients with MUD, which may recover after 3 months of MUD-specific therapy paving the way for further learning-based interventions. He L. et al. conducted a meta-analysis and indicated that brain-derived neurotrophic factor (BDNF) Val66Met polymorphism might be a potential genetic factor for MUD. Qi et al. investigated the relationship of insula cortex (IC) abnormalities among MA users with craving and whether IC predicts relapse susceptibility. Their study provides the first evidence that structural MRI may be used to diagnosis the craving state in MA users based on optimal cut-off values, which could be served as MRI bio-markers and an objective measure of craving state.

Schiffer et al. conducted a randomized, double-blind, placebo-controlled protocol, and suggested that unilateral transcranial photobiomodulation (t-PBM) to the hemisphere associated with positive emotional valence was an effective and safe treatment for opioid cravings as well as for depression and anxiety. Another study on opioids was conducted by Zhang et al. In this study, the conditioned place preference (CPP) paradigm was used for morphine-treated rats and saline-treated rats, and adopted 16S rRNA sequencing for the fecal bacterial communities at baseline and post-conditioning. This study provides direct evidence that morphine exposure alters the composition of the gut microbiota in rats and that microbial alterations are correlated to the sensitivity to morphine reward. These findings may help develop novel therapeutic and preventive strategies for opioid use disorder. Deng et al. conducted a randomized, vignette-based study, which aims to explore people's attitudes and beliefs toward methadone maintenance treatment (MMT), and stigmatization of MMT patients in China. They revealed that respondents showed a significantly higher level of stigma and discrimination toward the patient receiving MMT than the patient receiving amlodipine treatment, and suggested more efforts are needed to minimize the impact of MMT-related stigma to maximize MMT patients benefiting from MMT programs.

Li et al. conducted Morris water maze tests on rats to evaluate spatial learning and memory after 10 d of ketamine withdrawal. The current study suggested chronic ketamine exposure (25 mg/kg) causes spatial learning and memory deficits in rats, which may be associated with decreased serum BDNF levels. However, further explorations are needed to understand the relationship between ketamine and cognitive function and serum BDNF in human beings, and their underlying mechanisms.

### Benzodiazepine

Liu, Jian et al. conducted a systematic review and meta-analysis to determine the effects of benzodiazepine (BZD) in the elderly. They concluded that processing speed was significantly impaired in BZD users, and global cognition was significantly impaired in BZD abusers but not in BZD regular users. The same research group (Liu, Jian et al.) conducted a study of highly educated older adults to determine the effects of long-term use of BZD on cognition. They determined that BZD use may be significantly associated with worse executive functioning in this population. However, there was no association between the duration of BZD use and increased cognitive deficits. Both studies identified future experimental directions including potential longitudinal studies, within-subject studies comparing mood disorder patients' cognitive performance before and after the onset of BZD use, and between-subject studies that directly compare BZD's effect on subjects with the same baseline of cognitive functioning.

### Alcohol

Liu, Tian et al. addressed the association of metabolites in the frontal cortex and amygdala with cognitive deficits in alcohol dependent patients with aggressive behavior (AB-ADs). The results showed that metabolite alterations may be involved in the pathophysiology of AB in AD and its associated cognitive impairment, especially immediate memory and delayed memory. Cheng et al. explained the effects of early social environments and experiences on cognitive function in Alcohol Use Disorder (AUD) patients by comparing individuals with AUD and HCs. Their results suggest that cognitive function, especially social cognitive function, is affected by early life experiences. Cao et al. focused on cognitive impairment in AD patients, suggesting that the combined use of assessment of neuropsychological status and event-related potentials (ERPs) may serve as an objective basis for early diagnosis of cognitive impairment in patients with alcohol dependence. Yang K. et al. investigated group differences in cortical thickness in patients with alcohol dependence (PADs) and HCs and the relationship of cortical thickness to apathy, a feature of alcohol dependence. The results suggest the thickness of bilateral parietal and occipital temporal cortices as neural markers of apathy in PADs. These findings add to the literature by identifying the neural bases of a critical clinical feature of individuals with alcoholism.

Two studies examined resting state functional connectivity as a biomarker for relapse. Muller and Meyerhoff used two timepoints' rs-fMRI data from alcohol use disorder (AUD) individuals to investigate the degree to which relapse or abstinence in AUD treatment seekers affects the brain's intrinsic community structure. They found a significant reconfiguration of the extended brain reward system (eBRS) regions' involvement, which may be an early indicator for treatment failure in a subgroup of AUD patients. Yang, Meng et al. investigated AD- and relapse-associated functional connectivity (FC) of the nucleus accumbens (NAc) and medial prefrontal cortex (mPFC) with other brain regions during early abstinence. They found that FC between selected seeds and other reward- and/or impulse-control-related brain regions were associated with AD and relapse; these FC patterns could be potential biomarkers of AD or for prediction of relapse.

Huang H. et al. conducted a hospital-based survey on alcohol dependence (AD) patients to explore the relationship between AD and depressive symptoms. Their findings indicated high comorbidity between AD and depressive symptoms. Wang, Lu et al. conducted an online retrospective survey finding a slight reduction in alcohol consumption during COVID-19 in China; however, hazardous drinking is common, especially among male alcohol drinkers. Thus, public health policymakers should pay more attention to developing effective, population-based strategies to prevent harmful alcohol consumption.

### Tobacco

Cui et al. used event-related potentials (ERPs) to determine diminished neural signals related to error processing in young smokers after 12 h of abstinence, providing insight into the effects of short-term abstinence on neural measures of cognitive processing that may be useful for development of relapse prevention strategies. Xue et al. compared measures of dynamic resting state regional neural activity from 40 young smokers and 42 non-smokers and revealed that the pattern of variability was different between young smokers and non-smokers, indicating that dynamic regional indices might be a novel neuroimaging biomarker of smoking behavior in young smokers. Ye et al. used a multivoxel pattern analysis with a searchlight algorithm and SVM on structural MRI to identify the spatial pattern of gray matter volume in heavy smokers. Regions were primarily located at the temporal cortex, prefrontal cortex, occipital cortex, thalamus, insula (left), anterior and median cingulate gyri, and precuneus (left). Such findings might provide insights for understanding the mechanisms of chronic smoking and the development of effective cessation treatments. Xia et al. conducted a nationwide survey, finding that smoking was relatively higher among mental health professionals working in China who experienced burnout, sleep problems, and other factors relative those with higher education and who exercised regularly.

## Betel-Quid

To examine brain functional activity in the prefrontal cortex (PFC) in individuals with betel-quid dependent (BQD), Kong et al. recruited 48 participants with BQD and 22 normal controls (NCs). Both BQ-specific cue reactivity and Go/NoGo tasks were administered with functional magnetic resonance imaging (fMRI). They found disrupted function in PFC in individuals with BQD, which could be used as potential guidelines for diagnosis and treatment of BQD. Another study was conducted by Sariah et al. who found that BQD participants displayed less cortical thickness in the precuneus, entorhinal, right paracentral, middle temporal, and caudal middle frontal gyri compared to HCs, and that the severity of betel quid dependence is negatively associated with thickness in right caudal middle frontal cortex. These results indicate alterations in cortical thickness with BQD.

## Non-substance Addiction

### Internet Disorder or Internet Addiction (IA)

The COVID-19 pandemic has had a major impact on people's health all over the world. When it comes to addictive disorders, not only illicit substance use but also problematic Internet use have been reported to increase. These issues could be partly due to limited access to clinical services, but on the other hand, it is possibly a consequence of self-coping with anxiety and/or loneliness naturally produced in this very unusual situation. Dong et al. performed a self-report survey and showed excessive Internet use among children and adolescents, and Internet use is linked to depression and stress. Shan et al. measured the relationships between gender and other factors with Internet addiction (IA) in 1st year university students in South China. The results indicated gender differences IA severity in the students. Negative coping styles, acceptance of self and others, state anxiety levels had a significant association with severity of IA. Shen et al. investigated the prevalence and clinical correlates of insomnia in Chinese college students with IA. They found the prevalence of insomnia among students with IA was 54.86%. Compared with IA students without insomnia, IA students with insomnia were more likely to be younger, smoking, drinking, have anxiety, depression, suicidal ideations, suicide plans, and suicide attempts. Moreover, drinking, anxiety, and suicidal ideation were independently associated with insomnia in IA students. These three studies provide valuable evidence for school counselors and clinical professionals when facing students with IA.

### Internet Gaming Disorder (IGD)

Qin et al. suggested that the optimal cutoff score of the nine-item Internet Gaming Disorder Scale–Short-Form (IGDS9-SF) is 32 for the positive diagnosis of Internet Gaming Disorder (IGD) in a Chinese context. Xiang et al. explored the relationship between IGD and impulsivity and their study found that the prevalence of IGD among students in Guizhou province was 10.3%, and they also revealed the relationships between IGD and Behavioral Inhibition System (BIS), Behavioral Activation System-Fun seeking (BAS-F), BAS-Drive, and Barratt Impulsiveness Scale-11 (BIS-11) scores differed based on the age group of individuals tested. Liao et al. utilized a cross-sectional study to estimate the prevalence of IGD among Chinese adolescents and its association with their personality traits and Internet gaming characteristics. They revealed that personality traits and Internet gaming characteristics were significantly associated with IGD. Chen, Li et al. explored a study utilizing resting-state fMRI (rs-fMRI) in adolescents with IGD. This study confirmed the role of prefrontal-striatal circuits in the neural mechanism of IGD in adolescents. In the IGD group, bilateral cerebral medial orbitofrontal cortex (mOFC) synchronization was significantly reduced, which indicated that mOFC signal transmission in both hemispheres of the brain might be affected by impulse behavior and impaired response inhibition. Qiu et al. investigated the interaction between smoking and IGD on local spontaneous brain activity using ALFF based on rs-fMRI. Their findings indicate that smoking and IGD interacted with each other in the human brain, and may imply that people with IGD are tend to smoke, and smokers may be more vulnerable to Internet addiction than healthy people.

### Smart Phone Addiction

Because of the rapid development of communications and information technologies, smartphone use has been prevalent among young people, particularly university students. While smartphone use has made our lives more convenient, it may also have detrimental effects when overused. Huang Q. et al. conducted a cross-sectional survey and found that excessive smartphone use and mobile phone addiction are associated with poor sleep quality.

## Conclusion And Future Perspectives

To sum up, these articles provide findings that may facilitate development of effective evidence-based treatments for substance and non-substance addiction. These data are from the laboratory-based studies of addiction, school-based studies, and clinical and intervention studies of addicted patients across multiple fields. Multiple methodologies were used in their investigations, including rTMS, fMRI, s-MRI, rs-fMRI, and genetics, to explore the neural mechanisms, diagnosis, and treatment response of addiction. The results are helpful for further research in this field. Especially noteworthy with respect to treatment are the results of Schiffer et al., demonstrating a novel treatment that involves unilateral transcranial photobiomodulation of the hemisphere responsive to more positive valence (as indicated by the Dual Brain Psychology theory). Importantly, this treatment was effective and safe for reducing opioid cravings as well as for depression and anxiety.

Despite the extensive biomarkers of substance addiction and non-substance addiction reported here, more research is needed. Compared with substance addiction, research on non-substance addictions are relatively scarce. Behavioral addictions, such as IGD, IA, MUP, are worthy of more attention, for they affect not only adults but also children and adolescents.

## Author Contributions

YLiu has contributed to the conception and design of the Editorial. YLia, LL, and YW contributed considerably to the writing. DG, JC, KO, and WG contributed to the revision of the Editorial. All authors contributed to the article and approved the submitted version.

## Funding

This study was supported by National Natural Science Foundation of China (grant no. 81671325 to YLia), the Hunan Provincial Natural Science Foundation of China (grant no. 2020JJ4794 to YLia), the Japan Society for the Promotion of Science KAKENHI Grant-in-Aid for Young Scientists (grant no. 20K16634 to KO), Research Fellowship by Japan Research Foundation for Clinical Pharmacology (grant no. N/A to KO), Research Fellowship by Astellas Foundation for Research on Metabolic Disorders (grant no. N/A to KO), and the Naito Foundation Research Grant (grant no. N/A to KO).

## Conflict of Interest

The authors declare that the research was conducted in the absence of any commercial or financial relationships that could be construed as a potential conflict of interest.

## Publisher's Note

All claims expressed in this article are solely those of the authors and do not necessarily represent those of their affiliated organizations, or those of the publisher, the editors and the reviewers. Any product that may be evaluated in this article, or claim that may be made by its manufacturer, is not guaranteed or endorsed by the publisher.
